# Altered Expression of Glucocorticoid Receptor and Neuron-Specific Enolase mRNA in Peripheral Blood in First-Episode Schizophrenia and Chronic Schizophrenia

**DOI:** 10.3389/fpsyt.2020.00760

**Published:** 2020-08-12

**Authors:** Yong Liu, Yamei Tang, Cunyan Li, Huai Tao, Xiudeng Yang, Xianghui Zhang, Xuyi Wang

**Affiliations:** ^1^ National Clinical Research Center for Mental Disorders, and Department of Psychaitry, The Second Xiangya Hospital of Central South University, Changsha, China; ^2^ Department of Laboratory Medicine, The Second Xiangya Hospital, Central South University, Changsha, China; ^3^ Department of Laboratory Medicine, Hunan Provincial People’s Hospital, The First Affiliated Hospital of Hunan Normal University, Changsha, China; ^4^ Department of Biochemistry and Molecular Biology, Hunan University of Chinese Medicine, Changsha, China; ^5^ Department of Laboratory Medicine, The First Affiliated Hospital of Shaoyang University, Shaoyang, China

**Keywords:** first-episode unmedicated schizophrenia (FES), chronic schizophrenia, glucocorticoid receptor, GR transcripts containing exons 1B (GR-1B), cortisol, neuron-specific enolase, mRNA

## Abstract

**Introduction:**

It is well-known that altered hypothalamus–pituitary–adrenal (HPA) axis process has an important role in the neurodegenerative process in schizophrenia (SZ). However, this neurodegenerative mechanism has not been clarified in SZ. Therefore, the main purpose of this study was to determine HPA axis damage in the first-episode, unmedicated schizophrenia (FES) patients and chronic schizophrenia (CSZ) patients in comparison with healthy controls (HC) by means of quantitative analysis of the peripheral blood mRNA expression of glucocorticoid receptor (GR), GR transcripts containing exons 1B (GR-1B), and neuron specific enolase (NSE) genes and serum cortisol and NSE, a specific serum marker for neuronal damage.

**Methods:**

In the present study, 43 FES patients, 39 CSZ, and 47 HC were included. The peripheral blood mRNA expressions for GR, GR-1B, and NSE genes were determined by real-time quantitative polymerase chain reaction (RT-qPCR). Serum cortisol and NSE were analyzed by electrochemiluminescence immunoassay technique.

**Results:**

Levels of GR mRNA were significantly lower in FES and CSZ than that in HC. The expression of GR-1B mRNA was significantly decreased in CSZ when compared with that in FES. Levels of NSE mRNA were significantly lower in CSZ than that in FES patients or HC patients. CSZ patients showed significantly lower cortisol concentrations than FES and HC patients. FES patients showed significantly higher NSE concentrations than CSZ and HC.

**Conclusion:**

Our findings support that there is disrupted HPA axis system in the SZ and suggest that CSZ patients suffer a greater HPA axis damage than FES patients. Our research implicated underlying GR mRNA dysregulation in SZ and the potential importance of the functional GR-1B transcription in CSZ.

## Introduction

Schizophrenia (SZ) is a chronic severe neuropsychiatric disorder affecting almost 1% of the population worldwide (1). The neural diathesis stress model suggests that inappropriate and prolonged psychosocial stress can trigger or worsen the schizophrenic symptoms *via* the hypothalamus–pituitary–adrenal (HPA) axis (2, 3), which is a neuroendocrine system that mediates the stress response by secreting cortisol and maintains homeostasis in various physiological systems (4).

The glucocorticoid receptor (GR) is an important mediator of the maladaptive stress response by binding cortisol (5). Many researches showed decreased expression of total GR mRNA in the temporal cortex, in the dorsolateral prefrontal cortex (DLPFC), hippocampus, and amygdala in SZ and bipolar disorder (6–8). The human GR gene is a region of more than 80 kb within chromosome 5q31–q32, consisting of eight coding exons (exons 2–9) and nine 5′ non-coding first exons (exons A, B, C, D, E, F, H, I, J) (9, 10). Rodents and human GR levels are almost completely mediated at transcriptional level, and each non-coding exon variant is regulated by its own promoter, which is conducive to the tissue specificity of GR expression (10–13).

Indeed, an analysis suggested decreased GR transcripts containing exons 1B (GR-1B) mRNA expression in the DLPFC in SZ cases, and GR-1B mRNA levels accounted for 48% of variance in GR mRNA levels in the DLPFC (5). It is not known whether GR mRNA and protein abnormalities occurring in the orbitofrontal cortex (OFC), in the DLPFC, and/or in other brain regions in psychotic illness may also be found in the peripheral blood and to what extent GR expression patterns are found there.

Neuron-specific enolase (NSE) is an intracytoplasmic protein primarily localized in neurons and neuroendocrine tissue and is not actively secreted (14). Increased NSE in cerebrospinal fluid or blood indicates neuronal destruction or brain damage. NSE has been extensively regarded as a marker for neuronal damage in several mental disorders, such as meningeal hemorrhage, Guillain–Barre syndrome, the Creutzfeldt–Jakob disease, thrombosis (15–17). NSE is also used for auxiliary diagnosis of central nervous system (CNS) tumors and for damage evaluation after traumatic brain injury and cerebral ischemia (17, 18). Other studies found significant rising of NSE in sensory and temporal cortex of schizophrenics and rising of serum NSE concentrations in treatment refractory schizophrenics compared to healthy controls (HC) (19). Egan et al. (20) found first-episode, unmedicated schizophrenia (FES) patients had decreased concentration of NSE in the cerebrospinal fluid (CSF) when compared with those in chronic schizophrenia (CSZ) subjects.

Although altered HPA axis process is related to the pathogenesis of SZ, only limited studies have been focused on the systemic changes of mRNA and protein in FES and CSZ patients and the change of serum concentrations that is easily available in psychiatric patients. Moreover, several studies have produced conflicting results (14, 21–24).

Based on the above referred publications, the purpose of the present study was to determine whether CSZ patients suffer greater HPA axis abnormalities than FES patients. In order to address this aim, the expressions of GR mRNA and GR-1B mRNA in the peripheral blood and the serum concentrations of cortisol and NSE were quantified in FES, CSZ, and HC. We sought to: 1) determine whether expression levels of GR and GR-1B mRNA are altered in FES and/or CSZ cases compared to controls; 2) quantify the expression of serum concentrations of cortisol and NSE in FES and/or CSZ cases relative to controls, and 3) determine if selected GR and GR-1B mRNA are related to protein expression.

## Materials and Methods

### Subjects

43 FES patients, 39 CSZ patients, and 47 HC were recruited from the Department of Psychiatry in the Second Xiangya Hospital, Central South University. All the patients were diagnosed formally with SZ by two senior psychiatrists according to the *Diagnostic and Statistical Manual of Mental Disorders, Fifth Edition* (DSM-V) and evaluated using Positive and Negative Symptom Scale (PANSS) by a senior psychiatrist. There is no history of antipsychotics for CSZ patients for at least one month prior to study enrollment. All of the patients signed an informed consent before participating in this study. Patients were excluded from the study if they met one or more of the following criteria: comorbid mental disorders, other blood disease, a history of traumatic brain injury or intellectual disability, or cardiac–cerebral vascular disease.

### Analysis of Cortisol and NSE

Blood samples of participants were collected between 7.00 a.m. and 8.00 a.m. before food consumption. Serum cortisol and NSE were determined using an electrochemiluminescence immunoassay technique on a Cobas600 (Roche Diagnostics, IN, USA).

### RT-qPCR

10 ml peripheral venous blood samples were drawn from fasting FES, CSZ patients, and HC in the morning into EDTA tube. Peripheral blood mononuclear cells (PBMCs) were isolated and stored at −80°C until the RNA extraction.

The RNA was extracted from PBMCs in MagNA Pure LC2.0 Automatic extractor with MagNA Pure LC Total Nucleic Acid Isolation Kit–High Performance: automatic RNA extraction using magnetic beads (Roche Diagnostics, IN, USA). Complementary DNA (cDNA) was synthesized using Transcriptor First Strand cDNA Synthesis Kit (Roche Applied Science). Primers sequences were shown in **Table 1**. Each reaction contained 10 μl 2× SYBR Green Mastermix (Roche Applied Science), 1 μl of each primer pair (5 μM), and 5 μl of template cDNA in a 20 μl reaction volume. All reactions were performed with the Roche LightCycler 480 (Roche) using the LightCycler 480 SYBR Green I Master (Roche). The reaction mix was incubated at 95°C (10 min), followed by 40 cycles of 95°C (10 s), 60°C (10 s), and 72°C (20 s). A single fluorescence read was taken at the end of each 72°C step. Melting curve analysis controlled the specificity of the amplification. Reactions were performed in triplicate for each sample. The average value of the replicates for each sample was calculated and expressed as a cycle threshold (Ct). The housekeeper gene, *β*-actin and GAPDH were used as the internal control. The relative mRNA amounts of target genes were calculated by the 2^−ΔΔCt^ method.

**Table 1:** Primer sequences of the target genes and reference gene.

Gene	Primer sequences	Amplicon length (bp)
GR	F-CAGCTCCTCAACAGCAACAACA	139
	R-GTGCTGTCCTTCCACTGCTC	
GR-1B	F-CCGGGCCCAAATTGATATTCACT	205
	R-GTCTTCGCTGCTTGGAGTCTG	
NSE	F-GAACAGTGAAGCCTTGGAGCT	218
	R-TGGAGACCACAGATAGTCCC	
*β*-actin	F-TCCCTGGAGAAGAGCTACGA	136
	R-TGAAGGTAGTTTCGTGGATGC	
GAPDH	F-CGAGATCCCTCCAAAATCAA	170
	R-TTCACACCCATGACGAACAT	

### Statistical Analysis

Data were shown as mean ± SD for normal distribution variables (normality determined by Kolmogorov–Smirnov test). SPSS 18.0 software (version 18.0; Chicago, IL, USA) was used for statistical analysis. Categorical data were analyzed using the χ^2^ test. Data were analyzed by one-way ANOVA followed by Bonferroni *post hoc* corrected significance levels for comparison between each of the three groups. Spearman correlation coefficients were calculated for associations among variables. *P* < 0.05 was considered statistically significant.

## Results

### Demographic Characteristics

The demographic data of FES, CSZ patients, and HC were shown in **Table 2**. There were no significant differences in the mean age, gender among three groups (*P* > 0.05). FES and CSZ groups showed no significant between-group diﬀerences in PANSS (*P* > 0.05).

**Table 2:** Demographic data for FES, CSZ patients and HC.

	FES	CSZ	HC	*F* or*χ* ^2^	*P*
**Sex**	**N (%)**	**N (%)**	**N (%)**		
Male	27(62.8)	24(61.5%)	29(61.7)		
Female	16(37.2)	15(38.5%)	18(38.3)		
Total	43	39	47	0.017	0.992
Smoking				0.171	0.918
Yes	22	19	25		
No	21	20	22		
Age(Mean ± SD)	22.26 ± 4.49	24.10 ± 4.60	23.32 ± 2.68	2.971	0.055
Male	21.70 ± 3.24	23.34 ± 4.34	22.79 ± 2.73		
Female	23.19 ± 5.73	25.43 ± 3.98	24.11 ± 2.47		
PANSS					
Total	72.57 ± 20.50	72.20 ± 18.70	/	0.156	0.877
Positive	19.79 ± 4.97	18.1 ± 6.01	/	0.311	0.757
Negative	19.33 ± 6.85	21.08 ± 7.10	/	0.823	0.413
General	33.45 ± 9.77	32.90 ± 9.35	/	0.248	0.804

### Whole Blood Glucocorticoid Receptor mRNA Expression Levels

**Figure 1:**
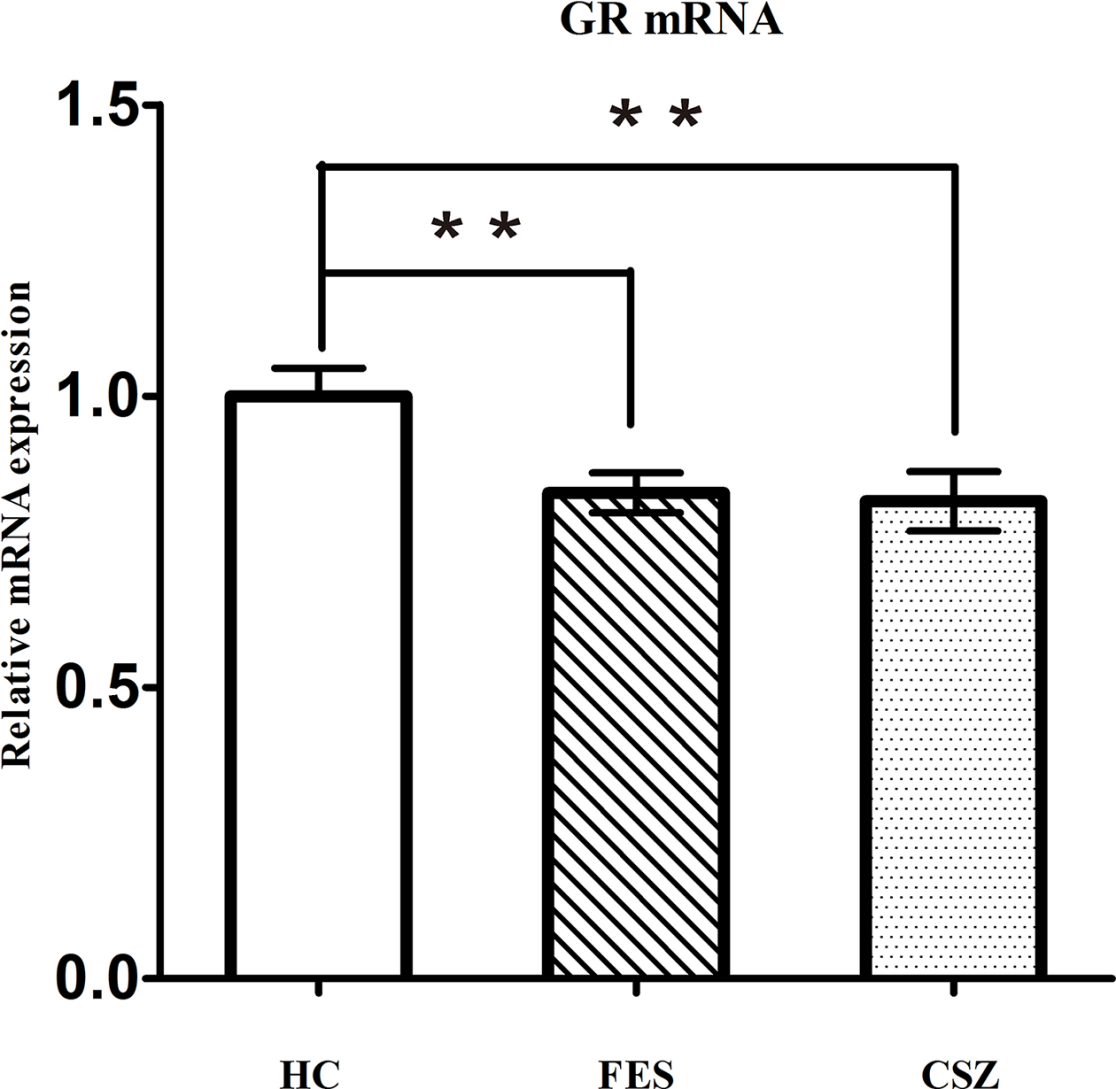
Representative of the relative mRNA expression of GR gene. ** means *P* < 0.01. Error bars denote mean and SD. FES, first-episode schizophrenia; CSZ, chronic schizophrenia; HC, healthy controls; GR, glucocorticoid receptor.

The semiquantitative evaluation of mRNA expression of GR genes was performed using real-time PCR, and *β*-actin was used as the internal control in peripheral blood. ANOVA analysis revealed significant difference among these three groups, for GR (F = 5.152, df = 128, *P* = 0.007). **Figure 1** showed that levels of GR mRNA were significantly lower (*P* = 0.008 and *P* = 0.005, respectively) in FES patients (0.834 ± 0.226) and CSZ patients (0.820 ± 0.318) than that in HC (1.000 ± 0.316).

### Human GR-1B mRNA Expression Levels

**Figure 2:**
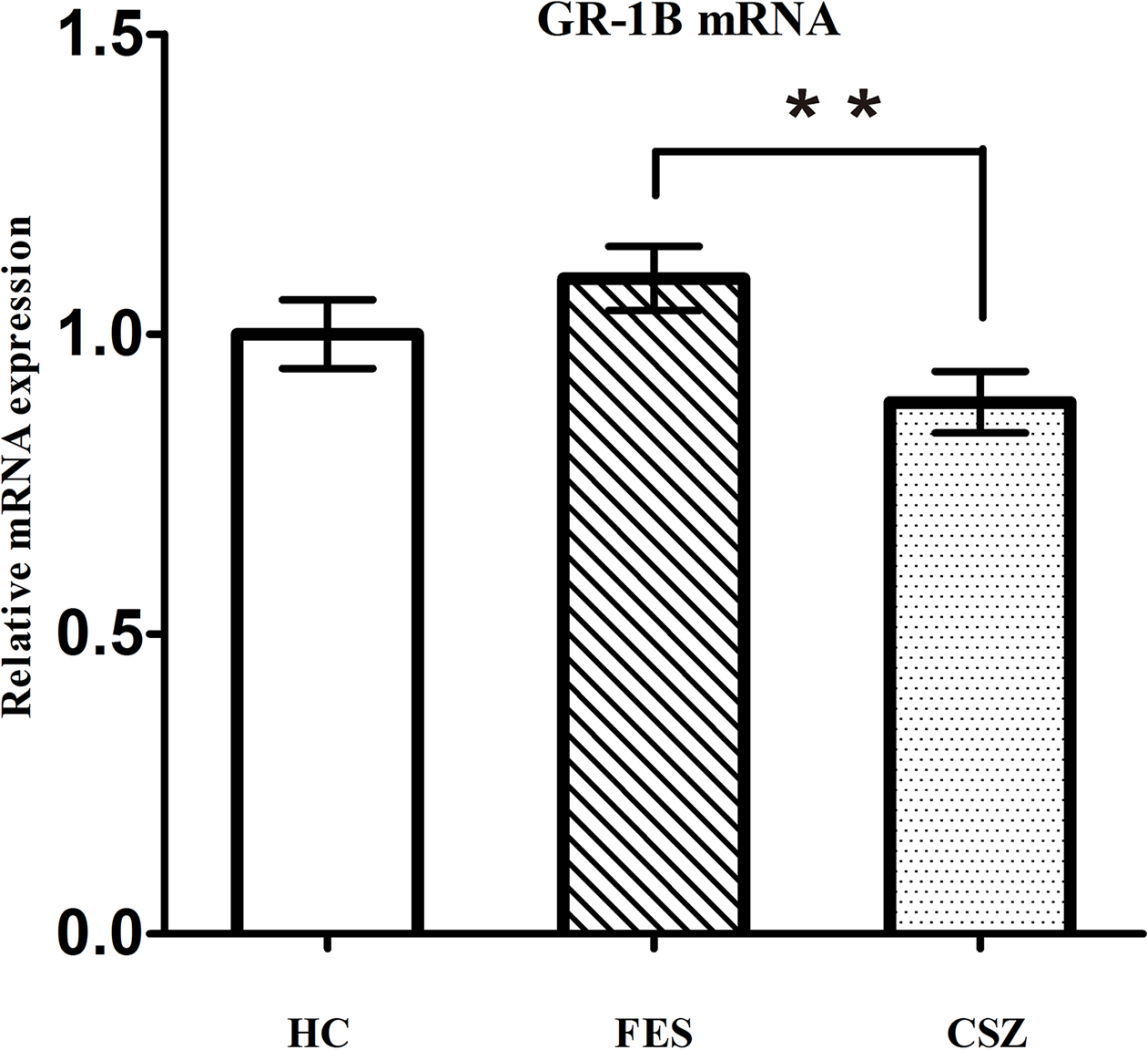
Representative of the relative mRNA expression of GR 1B gene. ** means *P* < 0.01. Error bars denote mean and SD. FES, first-episode schizophrenia; CSZ, chronic schizophrenia; HC, healthy controls; GR 1B, GR transcripts containing exons 1B.

ANOVA analysis revealed significant difference among these three groups, for GR-1B (F = 3.527, df = 128, *P* = 0.032). **Figure 2** showed that the expression of GR-1B mRNA was significantly decreased (*P* = 0.009) in CSZ patients (0.886 ± 0.319) when compared with that in FES (1.000 ± 0.381).

### Human NSE mRNA Expression Levels

**Figure 3:**
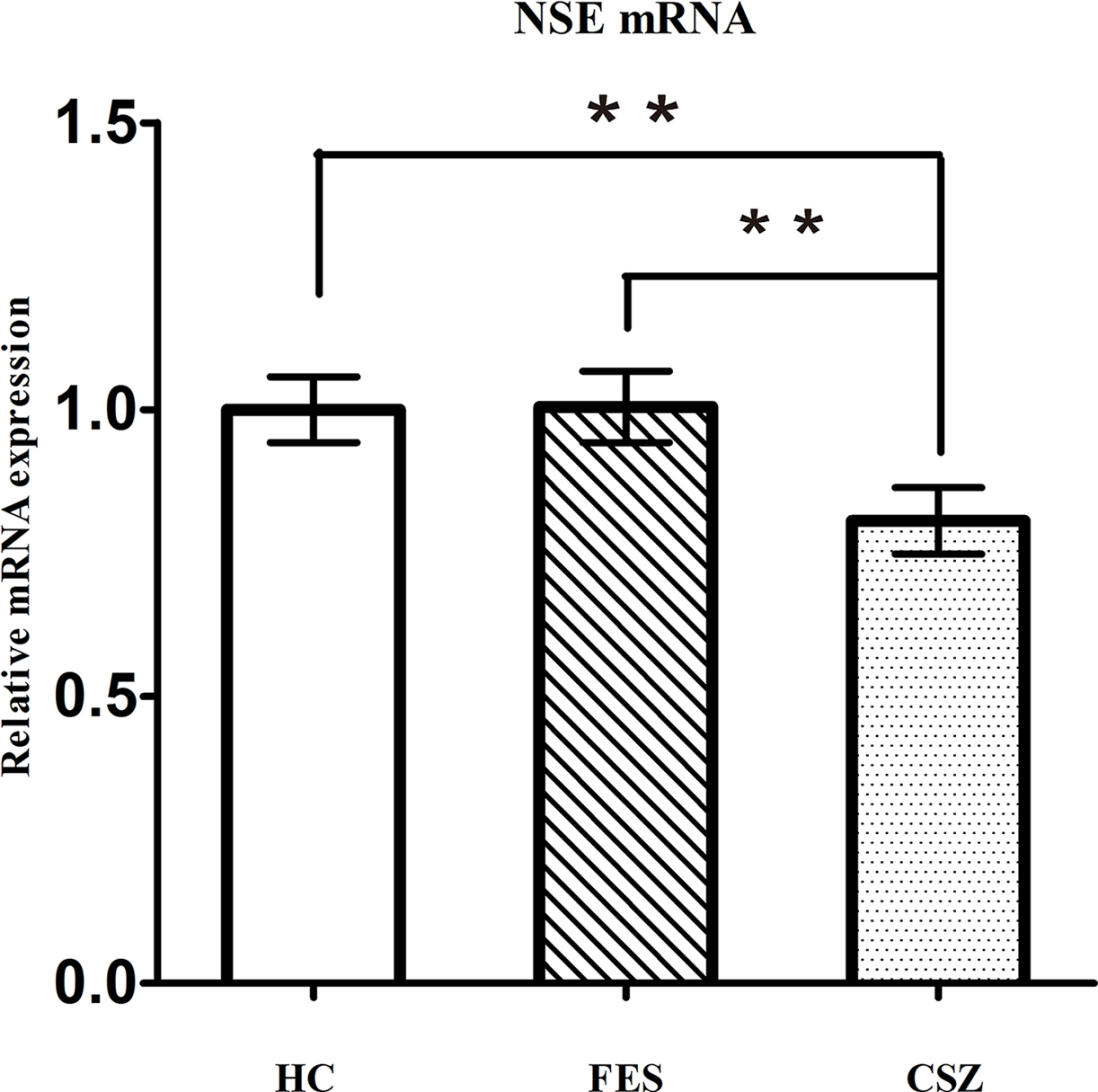
Representative of the relative mRNA expression of NSE gene. ** means *P* < 0.01. Error bars denote mean and SD. FES, first-episode schizophrenia; CSZ, chronic schizophrenia; HC, healthy controls; NSE, neuron-specific enolase.

ANOVA analysis revealed significant difference among these three groups, for NSE (F = 3.491, df = 128, *P* = 0.034). **Figure 3** showed that levels of NSE mRNA were significantly higher (*P* = 0.021 and *P* = 0.024, respectively) in FES patients (1.005 ± 0.414) and HC patients (1.000 ± 0.381) than that in CSZ (0.806 ± 0.362).

### Human Cortisol Expression Levels

**Figure 4:**
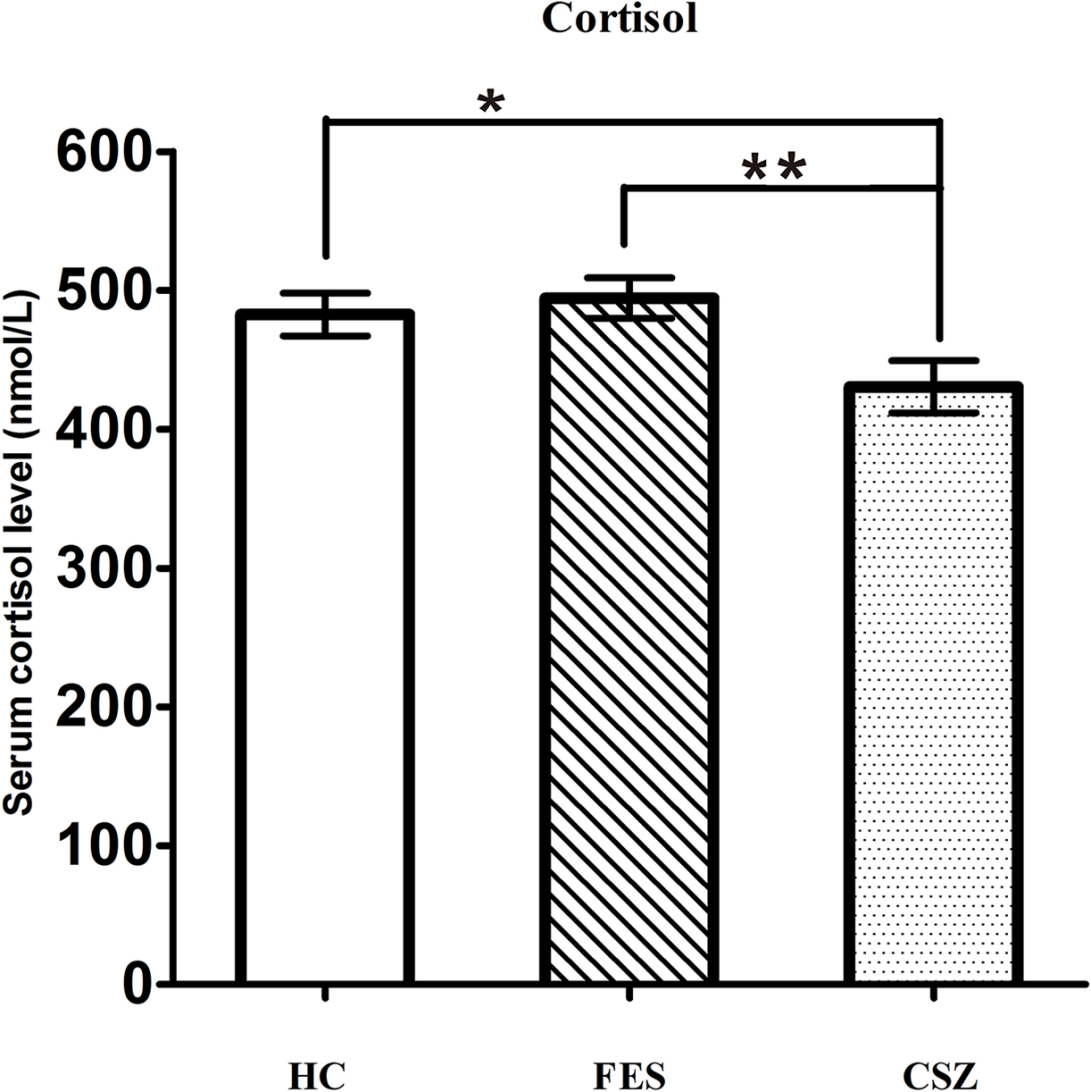
Representative of serum level of cortisol. * means *P* < 0.05, ** means *P* < 0.01. Error bars denote mean and SD. FES, first-episode schizophrenia; CSZ, chronic schizophrenia; HC, healthy controls.

ANOVA analysis revealed significant effects among these three groups for cortisol (F = 4.177, df = 128, *P* = 0.018). The serum level of cortisol in the CSZ group (430.51 ± 117.49 nmol/L) was significantly lower than that in the FES group (494.51 ± 95.22 nmol/L) (*P* = 0.007) or in the HC group (482.66 ± 105.69 nmol/L) (*P* = 0.025), while no significant difference was found in serum levels of cortisol between the FES group and HC group (*P* = 0.598) (**Figure 4**).

### Human NSE Expression Levels

**Figure 5:**
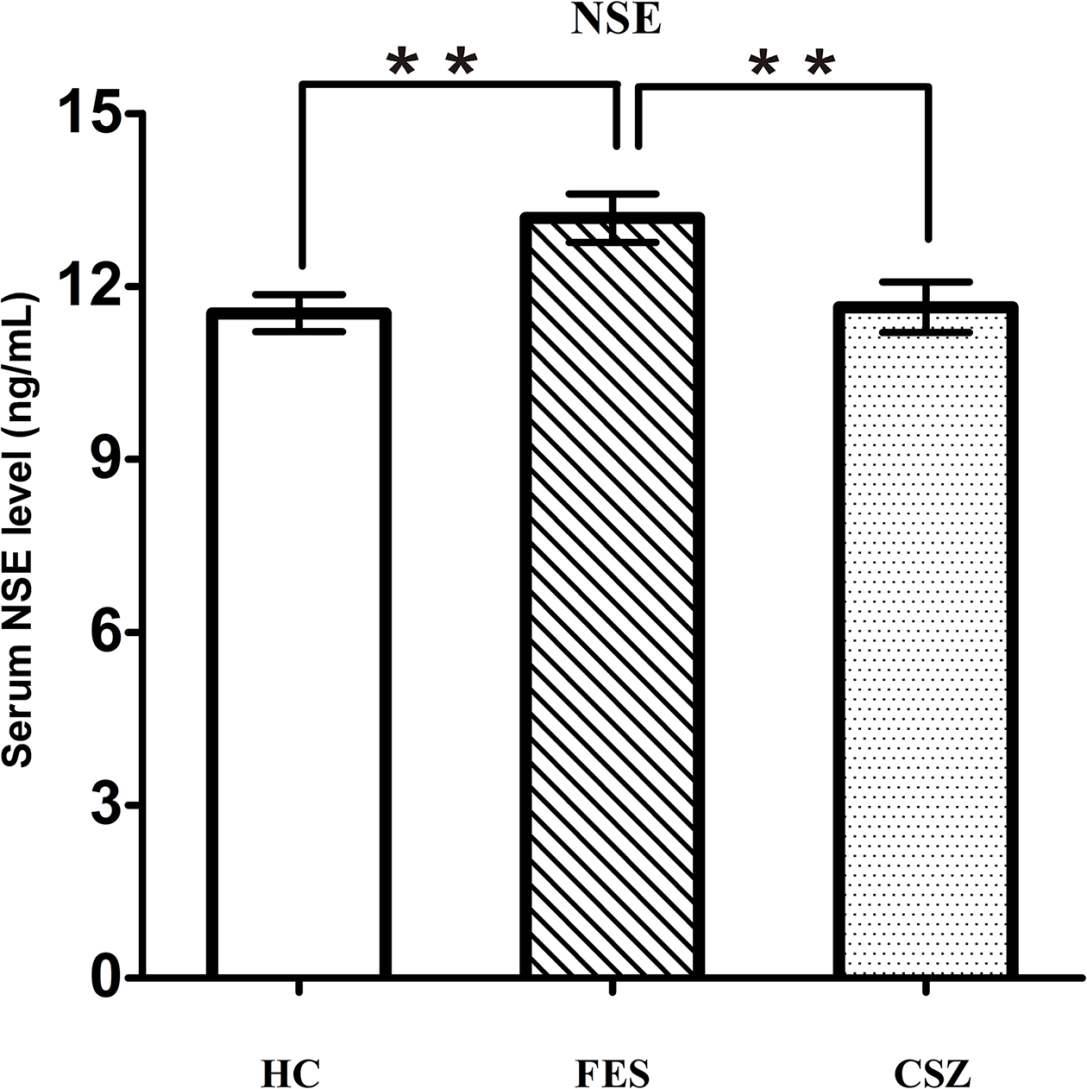
Representative of serum level of NSE. ** means *P* < 0.01. Error bars denote mean and SD. FES, first-episode schizophrenia; CSZ, chronic schizophrenia; HC, healthy controls; NSE, neuron-specific enolase.

ANOVA analysis revealed significant effects among these three groups for NSE (F = 5.644, df = 128, *P* = 0.004). Bonferroni *post hoc* test showed that the serum protein level of NSE in the FES group (13.18 ± 2.76 ng/ml) was significantly increased when compared with the HC group (11.54 + 2.20 ng/ml) (*P* = 0.003) or the CSZ group (11.63 ± 2.73 ng/ml) (*P* = 0.007), while no significant difference was found in serum levels of NSE between the CSZ group and HC group (*P* = 0.855) (**Figure 5**).

### The Relationships Between GR and GR-1B mRNA Transcription With NSE mRNA Levels and Serum Cortisol Level Related to Serum NSE Levels

We also assessed the relationships between GR and GR-1B mRNA transcription with NSE mRNA levels and serum cortisol level related to serum NSE levels using Spearman correlations. However, there were no statistically significant correlations of GR and GR-1B mRNA transcripts with NSE mRNA levels or serum cortisol level and serum NSE levels (*P* > 0.05).

## Discussion

In this study, we identified abnormal serum levels of cortisol in CSZ patients and NSE protein levels in FES patients. We provide further evidence of altered NSE mRNA in CSZ, GR mRNA in FES and CSZ and decreased GR-1B mRNA in CSZ compared to FES. These abnormalities particularly implicated the HPA axis disorder in SZ and down-regulated GR mRNA in SZ, decreased GR-1B mRNA transcriptional variant in CSZ. Our results also suggest that dysregulation of GR mRNA and protein expression arises in a different SZ stage.

Our results indicated the serum level of cortisol in CSZ patients was significantly lower than that in the FES group or in the HC group (*P* < 0.05). This concurs with other researches (22, 25–28). Rui Peng, et al. (22) reported that atypical antipsychotics could suppress the HPA axis activity by lowering cortisol levels in serum based on the measured levels between before and after treatment. However, another study showed that atypical antipsychotic treatment could contribute to increase serum levels of the cortisol (29). In addition, our findings also showed no significant difference in serum cortisol levels between the FES and HC groups (*P* > 0.05), which is consistent with previous researches (24, 30, 31), while some findings showed that CSZ patients had higher cortisol concentration (22, 29) or no elevated diurnal cortisol level in antipsychotic-naive, putatively at-risk children who present multiple antecedents of SZ or a family history of illness (23). Furthermore, dysfunctional HPA axis activities with reduced cortisol decline have been associated with poorer cognitive performance and memory deficits (25), poorer chronic response to daily stressors (failure to dampen during the day) (26), activation of dysfunctional dopamine pathways, and greater symptom severity (27, 28) in SZ patients. Taken together, these findings imply CSZ patients have more serious HPA axis dysfunctions than FES patients.

In accordance with most reported studies in the dorsolateral prefrontal cortex (DLPFC) (6, 23, 25–28, 32), we found that decreased GR mRNA expression in the peripheral blood was common to FES and CSZ, while decreased GR-1B mRNA expression was present only in CSZ. This may occur because other GR mRNA transcriptions including GR-1C (make up about 66% of total GR mRNA) may dilute the diagnostic effect (5). These results indicate that there is a significant imbalance in the expression of GR mRNA in different schizophrenic cohorts (7) and suggest GR-1B mRNA may be involved in the transcriptional regulatory mechanisms in CSZ governing GR-1B mRNA expression in CSZ. GR-1B mRNA transcript expression can be regulated by tissue-specific transcription factors (33, 34), GR promoter methylation (35), sequence variation in the GR gene promoter region in the human DLPFC (34). However, further research should be taken to verify and clarify the role of GR-1B expression in SZ.

However, our results showed that although GR mRNA was significantly reduced in both FES and CSZ, there were normal serum levels of cortisol in FES but reduced cortisol in CSZ. We hypothesize that feedback-regulated compensatory changes the activity of the *HPA* *axis* may cause an increased cortisol output in early stages of SZ, but this feedback compensation does not persist as schizophrenia continues over time in CSZ subjects (36). This may occur if GR-1B mRNA has an important role in regulating the expression of cortisol since this was decreased in CSZ and not FES.

In our present study, we only found increased serum NSE level in FES and no differences between CSZ patients and control subjects, while decreased NSE mRNA was found in CSZ patients. These results are in agreement with the study reporting increased serum levels of NSE in FES and normal serum levels of NSE in SZ (21). We speculate that CSZ patients have more serious neuronal destruction, which contributes to decrease of NSE mRNA in CSZ. The significant increase of serum NSE level in FES may owe to a greater neuronal disorder, which does not necessarily response to an active neurodegenerative process but to the failure of the neuronal energy mechanism that destroys membrane integrity and thus impacts its permeability (16). In addition, we observe neither correlation of NSE mRNA transcriptions with GR and GR-1B mRNA levels nor serum NSE level related to serum cortisol levels, suggesting that the change of NSE concentration has no direct correlation with HPA axis disorder, which may be regulated by other mechanisms.

## Limitations

There are certain limitations in the study. The first limitation is caused by the fact that we did not examine the impact of other possible risk factors on cortisol, such as rhythmic time, the potential role of antipsychotics and alcohol. In addition, the concentrations of these proteins and mRNA transcription before *versus* after antipsychotics treatment were not monitored. Last, this was a pilot study, the sample size in this study might not be large.

## Conclusion

Our findings of altered mRNA and protein expressions associated with HPA axis are consistent with many previous findings that SZ patients suffer HPA axis abnormalities. Our research also shows that some GR mRNA abnormalities found in the brains of patients with SZ can also be found in the mononuclear cells of living patients and may help specify that a decrease in GR-1B transcript subtype may be particularly important in the later stages of the disease characterized as chronic schizophrenia.

## Data Availability Statement

All relevant data is contained within the article. The original contributions presented in the study are included in the article/supplementary material, further inquiries can be directed to the corresponding author/s.

## Ethics Statement

This study was approved by the Ethics Committee of Second Xiangya Hospital, Central South University and was performed in accordance with the Declaration of Helsinki.

## Author Contributions

XW and YL designed the study. YT and XZ acquired the data, which XY and HT analyzed. CL wrote the article, which all authors reviewed. All authors contributed to the article and approved the submitted version.

## Funding

This work was supported by the National Natural Science Foundation of China (No. 81571307, 81771448, 81503276); the Hunan Provincial Natural Science Foundation of China (No. 2015JJ4069, 2018JJ2580, 2018JJ3387); the Hunan Provincial Science and Technology Bureau Foundation of China (No. 2017SK50509).

## Conflict of Interest

The authors declare that the research was conducted in the absence of any commercial or financial relationships that could be construed as a potential conflict of interest.

## Abbreviations

SZ, schizophrenia; CSZ, chronic schizophrenia; HC, healthy controls; HPA axis, hypothalamus–pituitary–adrenal axis; FES, the first-episode, unmedicated schizophrenia; GR, glucocorticoid receptor; GR-1B, GR transcripts containing exons 1B; NSE, neuron specific enolase; RT-qPCR, real-time quantitative polymerase chain reaction.
